# Sustainability in German radiotherapy: professionals’ perspectives on energy savings—results of a DEGRO working group survey

**DOI:** 10.1007/s00066-026-02544-x

**Published:** 2026-06-22

**Authors:** Ann-Katrin Exeli, Andreas Lurtz, Daniel Medenwald, Linda Agolli, Daniel Habermehl

**Affiliations:** 1 Department of Radiotherapy, Wilhelm-Conrad-Roentgen-Clinic, Justus-Liebig University Giessen, University Hospital Giessen-Marburg, Giessen, Germany; 2 Justus-Liebig University Giessen, Giessen, Germany; 3 Department of Radiation Oncology, University Hospital Magdeburg, Leipziger Straße 44, Magdeburg, Germany

**Keywords:** Radiotherapy, Hypofractionation, Ultrahypofractionation, Environmentally radiation oncology, Carbon footprint

## Abstract

**Purpose:**

The healthcare sector is a major source of carbon dioxide (CO_2_) emissions, with radiotherapy (RT) being a particularly significant contributor due to its high energy consumption and patient-related travelling. Our survey aimed to gather opinions on energy savings and CO_2_ reduction in various RT areas. We asked radiation oncologists and medical physicians about the importance of these issues in their day-to-day work as well as about their personal views on them.

**Methods:**

An online survey was conducted via the German Society for Radiooncology (DEGRO) mailing list. The questionnaire covered demographics, attitudes toward energy-saving measures, and current clinical use of (ultra-)hypofractionation (UHF). Quantitative data were analyzed descriptively; free-text responses were thematically summarized.

**Results:**

A total of 69 questionnaires were completed. Attitudes toward sustainability in RT were largely positive: almost 80% believed that topics such as climate protection and energy transition could influence future departmental decisions, and 77% considered sustainable manufacturing and maintenance by linear accelerator providers important. Over 95% reported using UHF regimens. Moderate hypofractionation was the standard for breast (77%) and prostate cancer (55%), while longer schedules remained common for bone metastases (62%). Moreover, 58% indicated willingness to expand UHF use, provided no medical disadvantages occurred.

**Conclusion:**

There are broad awareness and support for sustainability in RT among German professionals. However, practical implementation of environmentally beneficial measures, such as UHF, remains constrained. Addressing systemic barriers and improving access to environmental impact data may facilitate more sustainable clinical workflows.

**Supplementary Information:**

The online version of this article (10.1007/s00066-026-02544-x) contains supplementary material, which is available to authorized users.

## Introduction

The healthcare sector is a significant contributor to global carbon dioxide (CO_2_) emissions, with radiotherapy (RT) playing a particularly important role due to its high energy consumption [1, 2]. As concerns about climate change and sustainability continue to grow, reducing the environmental impact of medical treatments and patient travel has become an increasingly relevant topic [3]. This very subject is being addressed by newly founded working groups of the national and international professional societies commonly used in RT, such as the DGMP (Deutsche Gesellschaft für Medizinische Physik), DEGRO (Deutsche Gesellschaft für Radioonkologie), or ESTRO (European Society for Radiotherapy and Oncology). In addition, the new Society Matters section, as an initiative from the Green Journal, will focus on themes related to sustainability, education, and healthcare policy [4]. Radiotherapy departments, which rely on energy-intensive equipment such as linear accelerators (linac) and on-board imaging systems, offer substantial potential for reducing energy consumption and the carbon footprint. Recent analyses have quantified the carbon footprint of external beam RT to range from 185 to 2066 kg CO_2_ equivalents per treatment course, with primary sources including manufacture and maintenance of the linac, patient and staff transportation, and facility energy use. Patient transport is a particularly notable contributor: a multicenter study from Germany reported average travel distances of 37.2 km per session, resulting in approximately 156 kg CO_2_ equivalents per full treatment course [5]. A previous study of our group showed that 90% of our patients came with their own car, while the average distance travelled per day to and from the clinic was 57.7 km. Switching from normofractionation to ultrahypofractionation (UHF) reduced CO_2_ emissions by up to 82%, depending on the irradiation region, as fewer patient visits were required [6].

Although sustainability is important in healthcare, it is unclear to what extent medical professionals consider energy efficiency and CO_2_ reduction in their daily practice. Therefore, it is essential to understand their perspectives in order to develop targeted strategies for implementing more sustainable practices without compromising the quality of patient care.

This survey aims to assess radiation oncologists’ and medical physics experts’ opinions on energy savings and CO_2_ reduction in their daily work. We aim to determine how relevant these issues are in their clinical routine and what their personal views are towards environmental responsibility in medical practice. By gaining insight into their views, we hope to identify potential barriers and opportunities for integrating more sustainable workflows into RT, thereby contributing to ongoing discussions about more environmentally friendly healthcare solutions.

## Materials and methods

The survey was designed by radiation oncologists and medical physics experts from the new DEGRO working group on sustainability in RT. The questions were developed specifically for the purpose of this survey and were asked in German. The questionnaire was developed specifically for this project and internally reviewed prior to distribution; however, it was not formally pretested or externally validated. All participants took part in the survey anonymously and on a voluntary basis. Participation was possible online via a link or QR code via the website www.allcounted.de. The survey was opened on 1 December 2024, and a call for participation was sent out via the DEGRO mailing list (1269 addressees) on 4 December 2024. The survey was closed on 28 January 2025.

The questionnaire (see the supplemental material for the original questionnaire as well as the English translation) consisted of 25 questions. The questions were either multiple choice, or individual text answers could be provided. The questions were subdivided into three categories:

- *demographic characteristics of the participants*: seven questions
- *opinion of the participants towards energy reduction in general and in their department*: eleven questions
- *currently used fractionation schemes in their department*: seven questions

The fractionation schemes provided in this questionnaire are summarized in Table 1.

**Table 1:** Overview of the standard, hypo-, and ultrahypofractionation schemes used in the questionnaire for breast cancer, prostate cancer, and bone metastasis.

	Breast	Prostate	Bone metastases
Standard fractionation	25–30 fx	35–40 fx	10–20 fx
Hypofractionation	15–16 fx	20–28 fx	5–6 fx
Ultrahypofractionation	5 fx	5–7 fx	1–5 fx

*fx* fractions

## Results

A total of 69 radiation oncologists and medical physicists completed the survey, which corresponds to a response rate of 5.4% (69/1269).

### Demographic characteristics of the participants

**Fig. 1:**
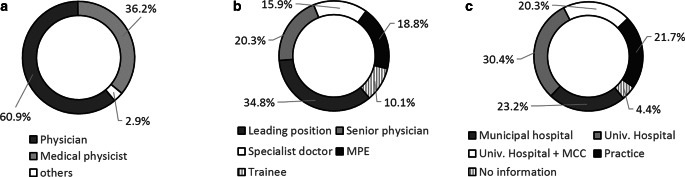
Pie charts showing demographic characteristics of the participants. **a** Distribution of respondents by professional group; **b** distribution of respondents by position; **c** working environment of the participants (*Univ.* university, *MCC* medical care center, *MPE* medical physics expert)

The age, gender, occupational group, and position of the participants are shown in questions 1–4 in the supplemental material. Almost 60% of the participants were between 40 and 59 years old and were male. The majority of respondents were medical doctors (61%), with medical physicists being the second most common group (36%; Fig. 1a). Among the participants, people in managerial positions were highly represented, with 35% belonging to the group of clinic directors, senior medical physicists, practice owners, or practice partners (Fig. 1b). Over 70% of participants work in a large city (> 100,000 inhabitants), most of them (30%) at a university hospital (see questions 5 and 7 and Fig. 1c).

### Opinion regarding the importance of sustainability

**Fig. 2:**
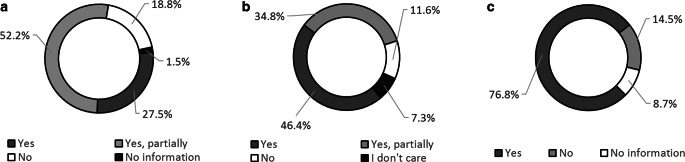
Pie charts displaying the mindset regarding the relevance of sustainability. **a** Do you think that the topics of “climate protection,” “energy transition,” and “sustainability” can influence clinical decisions in your department in the future? **b** Would you welcome the inclusion of such topics in everyday medical and radiotherapy practice? **c** Is it important to you that linear accelerator manufacturers produce sustainably or that the service/maintenance work demonstrates sustainable aspects?

When asked whether their employers had initiated campaigns focused on climate protection or energy-saving measures, 55.1% of respondents confirmed the existence of such initiatives, while 36.2% reported that no such efforts were in place and 8.7% stated they were not informed (see question 8 in the supplemental material). Among those aware of campaigns, only a minority reported active participation. Regarding the potential influence of climate protection, energy transition, and sustainability on future clinical decision-making, a significant majority (52.2%) believed that these topics would at least partially impact decisions within their department, with 27.5% anticipating a clear influence (Fig. 2a). Only 18.8% saw no relevance of these topics to clinical decisions. When asked whether they would support or promote the integration of sustainability-related topics into routine medical and RT practice, 46.4% responded affirmatively, and an additional 34.8% expressed partial support (Fig. 2b). Only a small portion of respondents (11.6%) opposed such integration, while 7.3% indicated indifference.

Finally, in estimating the daily energy consumption of the linac, 55.1% of respondents assumed a usage between 100 and 1000 kWh per day, which represents an appropriate estimate. None of the respondents estimated usage below 1 kWh per day, underscoring the substantial energy demands associated with RT equipment. The participants showed good intuition in the estimation questions on the subject of energy consumption (see question 12 on energy consumption of the linac, question 23 regarding the estimation of the extent to which normofractionation increases the CO_2_ footprint compared to UHF, and question 25 how much CO_2_ can be saved by a hypofractionation concept in the supplemental material).

Respondents demonstrated a positive attitude toward sustainability. A total of 53 of the 69 participants stated that it is important to them that linac manufacturers produce sustainably or that service and maintenance work has sustainable aspects (Fig. 2c). For almost half of the 69 participants, this was also a criterion when purchasing a new linear accelerator. When creating and optimizing radiation plans, 40 participants also stated that they pay attention to an electricity- and time-saving plan variant. Furthermore, this is further supported by the fact that only 3 participants assessed current developments towards more climate-neutral processes in the healthcare sector (“DGMP goes green,” “ESTRO Climate Change Awareness and Action”) negatively.

### Utilization of (ultra)hypofractionation schemes in clinical practice

**Fig. 3:**
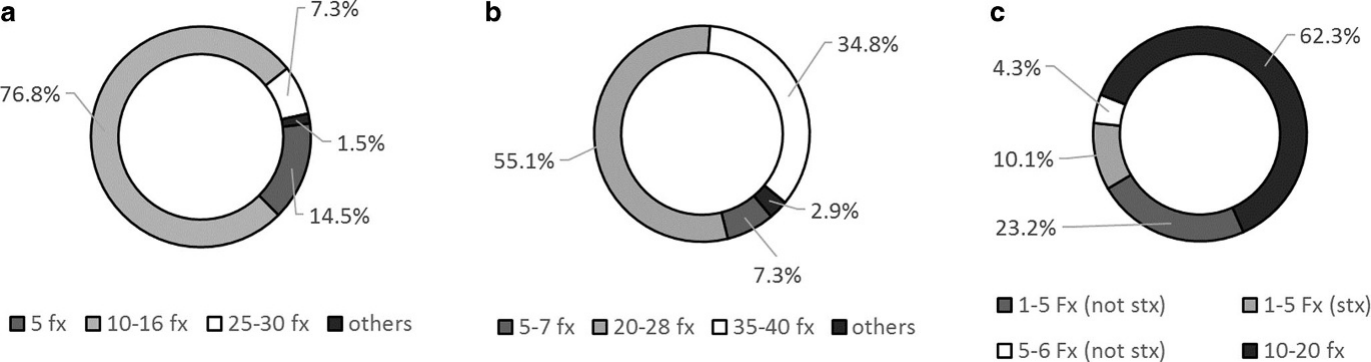
Respondents were asked to indicate the current standard fractionation scheme used in their institution for **a** breast cancer, **b** prostate cancer, and **c** bone metastases. The fractionation concepts were divided into standard, hypo-, and ultrahypofractionated concepts (*fx* fractions, *stx* stereotactic)

In general, almost two thirds stated that they use UHF concepts (single dose of 5 Gy or more) in their department. The reasons given for this were medical (96%), organizational (46%), and for reduction of energy consumption (16%; multiple answers were allowed). For the treatment of breast cancer without the use of a sequential boost or irradiation of the lymphatic drainage, a hypofractionated (HF) concept with 15 to 16 fractions represented the clinical standard for 77% of those surveyed. Only a fraction of the facilities (14.5%) used the shorter UHF concept with five fractions, while normofractionation with 25 to 30 fractions accounted for no more than 10% as the clinical standard (Fig. 3a). For prostate cancer treatment (without inclusion of the pelvic lymphatic drainage), 55% named an HF concept as their standard and 7% the UHF concept as the current standard in their department (Fig. 3b). The normofractionated or standard concept with 10–20 fractions was used most frequently (62%) for bone metastases, followed by UHF RT with 1–5 fractions (stereotactic: 10%, non-stereotactic: 23%; Fig. 3c).

### Summary of participants’ free-text comments

#### Technical measures and clinical integration

Many respondents highlighted the importance of technical and infrastructural improvements, including building efficiency, optimized lighting and heating systems, and the reuse of waste heat from medical devices. Common suggestions included the adoption of renewable energy sources, reduction of standby power, and implementation of automatic shutdown protocols. Several participants emphasized the role of digitization in both reducing resource consumption and in improving workflow efficiency. Clinically, sustainability was linked to evidence-based treatment approaches, particularly the HF concept and avoidance of unnecessary treatment fractions. Environmentally conscious procurement and device usage were also discussed.

#### Ethical considerations and clinical priorities

A prevailing view was that sustainability efforts must not compromise medical quality or patient care. While many participants supported integrating ecological responsibilities into clinical practice, concerns were raised about potential trade-offs between energy-saving measures and optimal treatment delivery. Nonetheless, the initiative behind the survey was widely welcomed, with respondents acknowledging its relevance and expressing appreciation for raising awareness within the field.

#### Barriers, criticism, and divergent views

Critical perspectives included concerns about the survey’s framing, potential loss of anonymity, and skepticism about the feasibility of implementing sustainability measures given current financial and regulatory constraints. Some respondents questioned the relative impact of energy savings in the context of high-cost treatments and broader healthcare priorities. A minority expressed ideological reservations or rejected the relevance of sustainability within radiation oncology altogether. Calls for structural changes—such as digital-only distribution of professional publications and increased energy autonomy for departments—reflect broader system-level considerations.

## Discussion

In recent years, the intersection of climate change and healthcare has gained significant attention, with increasing recognition of the healthcare sector’s substantial contribution to global greenhouse gas emissions. In this context, RT, as an energy-intensive discipline, has come under particular review. Within radiation oncology, recent analyses by Lichter et al. have provided a comprehensive overview of potential strategies for decarbonizing RT services, ranging from technical innovations to workflow optimization and fractionation adjustments [3].

Our survey provides insights into the current views, knowledge, and practices of radiation oncology professionals in Germany regarding sustainability and energy efficiency. While the limited sample size restricts generalizability, the responses reflect a professional community with a high level of awareness of climate-related issues and a generally positive attitude toward implementing environmentally conscious practices in their clinical workflows. However, the relatively low response rate and the overrepresentation of respondents in managerial positions and from large urban centers limit the generalizability of these findings and should be considered when interpreting the results.

Over half of the participants indicated involvement in climate protection activities, provided these were supported by their institutions. Moreover, a strong majority expressed openness to incorporating sustainability into future clinical decision-making. Nevertheless, specific knowledge gaps remain. For instance, although energy-saving modes such as “standby” or “sleep mode” were frequently cited as desirable interventions, most participants were unaware of whether such features existed or were actively used on their department’s linacs. Our own measurements confirmed that linacs consume substantial amounts of electricity even in standby mode, underscoring the need for technical transparency and improved staff education.

The number of treatment fractions was frequently discussed as a key determinant of radiotherapy’s environmental impact. This aligns with findings from Bedir et al., who quantified the CO_2_ emissions associated with varying fractionation regimens and found UHF concepts to offer significant reductions in emissions [5]. Similarly, it was demonstrated that adopting UHF protocols in common indications such as breast and prostate cancer and bone metastases could markedly reduce the carbon footprint of treatment without compromising clinical efficacy [6–10].

Despite the strong evidence base supporting UHF strategies in selected disease sites, our results reveal a discrepancy between knowledge and practice [7–11]. While many respondents reported using UHF protocols in their departments, only a minority routinely applied them to breast (10/69) or prostate cancer (5/69) cases. Instead, moderately HF (15–16 fractions) or normofractionated concepts (≥ 25 fractions) were still dominant, particularly for prostate cancer. This implementation gap is likely multifactorial. First, differences in guideline implementation and local clinical routines may contribute, as institutional standards and established workflows often shape treatment decisions. Second, ongoing concerns regarding toxicity and long-term oncological outcomes may limit broader adoption, despite growing evidence supporting HF schemes. Third, organizational and workflow-related barriers—such as scheduling, resource allocation, and departmental logistics—may further hinder implementation. In addition, economic disincentives and current reimbursement structures may discourage the use of shorter treatment regimens, as they are often less financially favorable for institutions [5]. Finally, a general reluctance to deviate from established treatment pathways may also play a role. The evidence for UHF is well established—as demonstrated by the recently published 10-year results from the FAST-Forward trial—offering improved patient comfort and contributing to more climate-friendly RT [8, 12].

This is at odds with the ecological benefits of (U)HF, which can significantly reduce patient travel—a major source of CO_2_ emissions in radiation oncology. Moreover, fewer treatment sessions may ease the financial burden on patients by lowering indirect costs such as transport and time off work, thereby reducing financial toxicity [13]. In this context, recent geospatial analyses of radiation oncology centers in Germany have highlighted the unequal distribution and regional vulnerability of access to RT services, demonstrating that longer travel times may become a critical factor for patients, particularly in rural areas [14]. Addressing these systemic misalignments is essential for making sustainable care both feasible and equitable.

The ongoing hospital reform in Germany may further increase the relevance of resource-conscious RT concepts [5, 6, 14]. While increasing centralization of specialized care could lead to longer travel distances for some patients, the broader push toward efficiency should also favor evidence-based shorter fractionation schedules that reduce both resource use and patient-related emissions. For indications with robust evidence supporting hypofractionation, such as breast cancer, prostate cancer, and palliative treatment settings, reimbursement structures should avoid maintaining financial incentives for unnecessarily prolonged treatment courses. Recent radiation oncology health-economic analyses suggest that fraction-based reimbursement may disincentivize the implementation of evidence-based hypofractionated schedules, whereas structural reimbursement reform toward value-based or bundled payment models may better align financial incentives with efficient, patient-centered care [15].

It is also worth noticing that clinicians may underestimate the actual environmental impact of RT or lack access to reliable data. Transparent and comparable reporting of linac energy usage, departmental emissions, and patient travel logistics could help practitioners to make more informed decisions. As highlighted in the literature, digital platforms could play a key role in promoting awareness and disseminating sustainability-related information among professionals and the broader public, thereby supporting more environmentally conscious practices in RT [16].

## Conclusion

Our survey reveals a positive attitude toward energy conservation and sustainable practices among the participants, including openness to UHF where clinically appropriate. The low response rate and overrepresentation of managerial and urban respondents limit the generalizability of the findings and should be considered in their interpretation. Despite its proven potential to reduce CO_2_ emissions, the implementation of UHF might be limited due to structural and informational barriers. Promoting education, transparency, and aligned incentives could help advance sustainability in radiation oncology.

## Supplementary Information

ESM1: Supplementary material 1

## Data Availability

All data generated or analyzed during this study are included in this published article and its supplementary information files.
