# Supplementary material for: Sustainability in German radiotherapy: professionals’ perspectives on energy savings—results of a DEGRO working group survey
Source: Strahlenther Onkol. 2026 Jun 22;202(8):803–8. doi: 10.1007/s00066-026-02544-x (PMC13391738; doi:10.1007/s00066-026-02544-x)
Supplement: Supplementary file 1 — ESM1: Supplementary material 1 [file 66_2026_2544_MOESM1_ESM.docx]

**Appendix**

Below is a list of all the questions from the survey in German and English, along with the corresponding answers. For estimation questions, the correct answer option is underlined in the analysis.

**Question 1: Which age group do you belong to? (“Welcher Altersgruppe gehören Sie an?")**


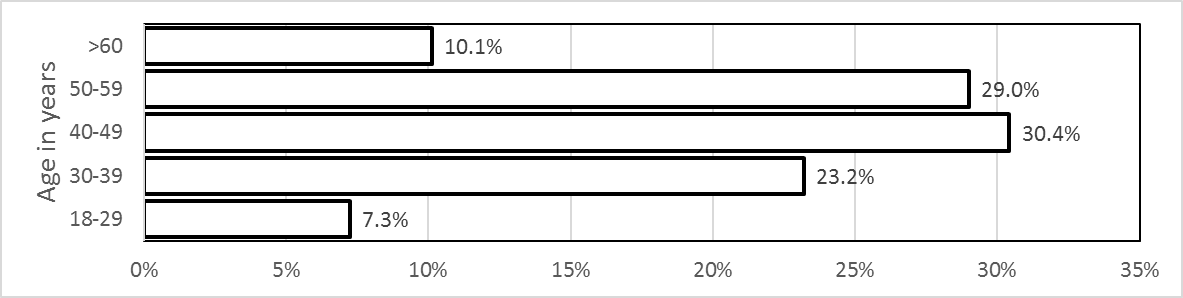


**Question 2: How do you identify yourself regarding your gender? ("Wie identifizieren Sie sich in Bezug auf Ihr Geschlecht?")**


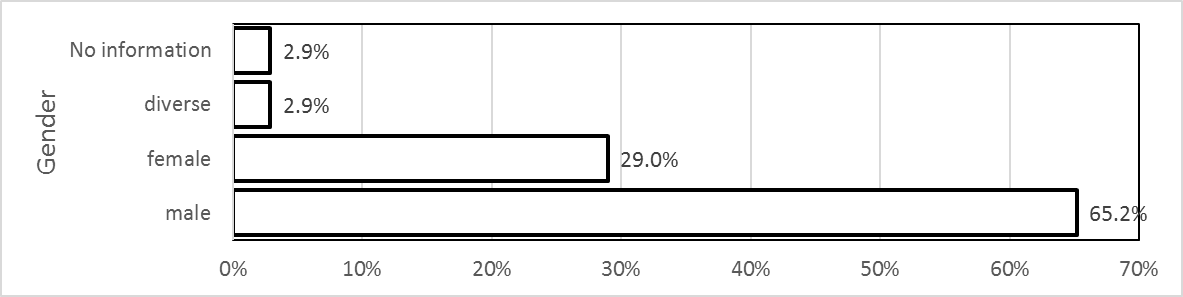


**Question 3: Which professional group do you belong to? ("Welcher Berufsgruppe gehören Sie an?")**


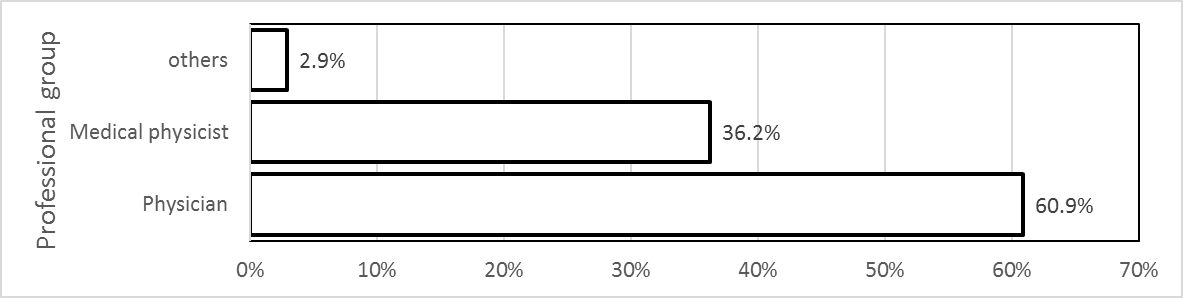


**Question 4: What position do you hold? ("In welcher Position sind Sie tätig?")**


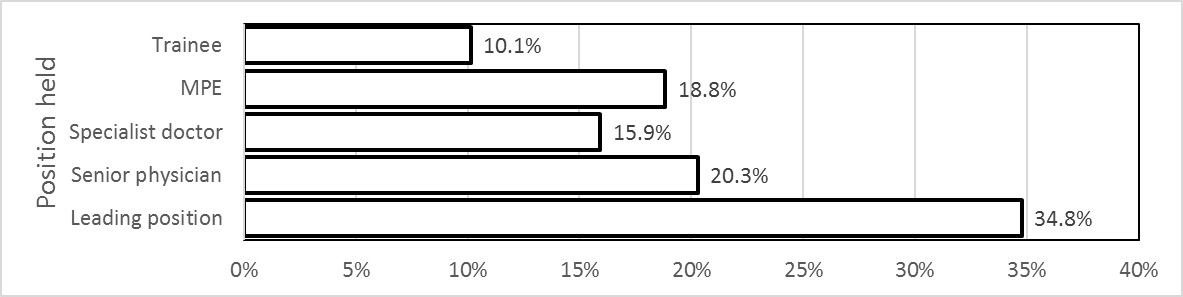


**Question 5: In which urbanization region do you work? ("In welcher Urbanisierungsregion arbeiten Sie?")**


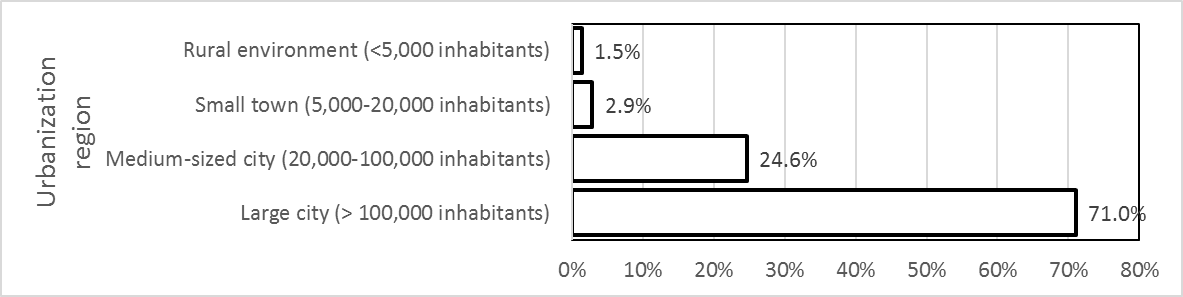


**Question 6: In which federal state do you work? ("In welchem Bundesland arbeiten Sie?")**


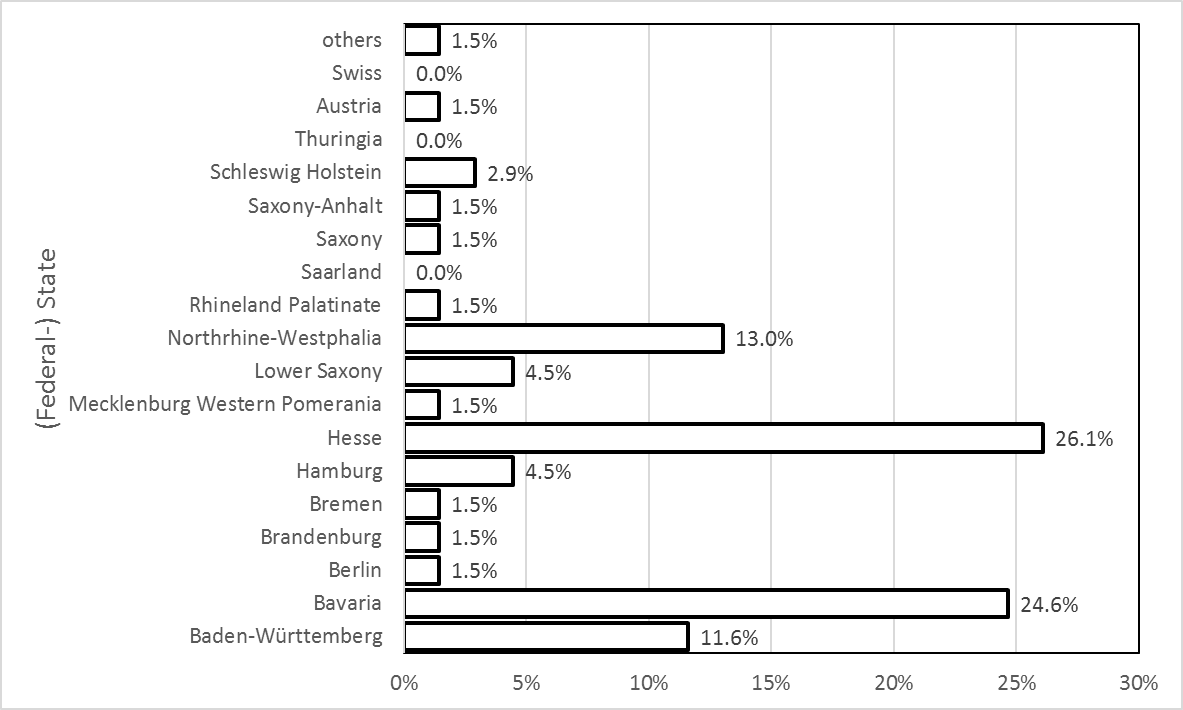


**Question 7: In which working environment do you work? ("In welchem Arbeitsumfeld sind Sie tätig?")**


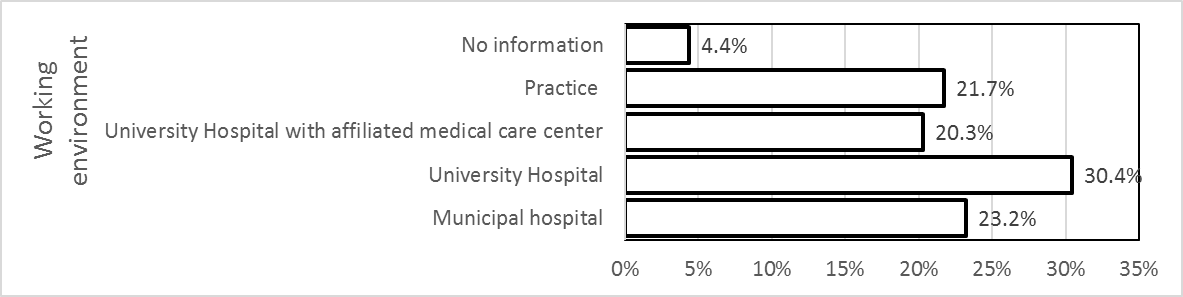


**Question 8: Does your employer initiate campaigns for climate protection or energy-saving measures? ("Gibt es von Ihrem Arbeitgeber initiierte Aktionen zu Klimaschutz- oder Energiesparmaßnahmen?")**


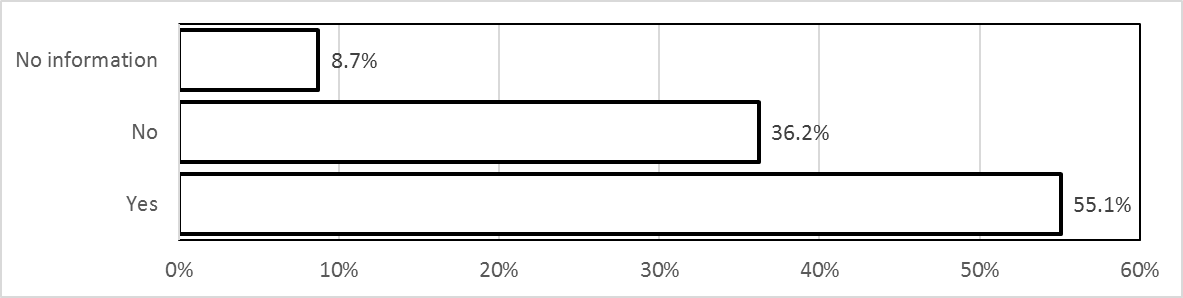


***Additional to Question 8: If yes, did you participate in this campaign? („Falls ja, haben Sie sich an dieser Aktion beteiligt?“)***


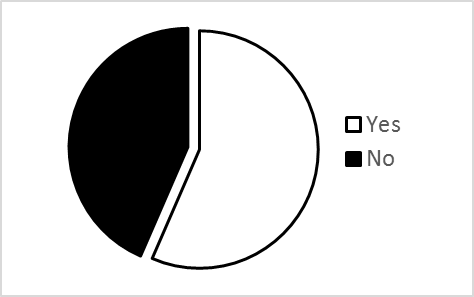


**Question 9: Do you think that the topics of "climate protection", "energy transition" and "sustainability" can influence clinical decisions in your department in the future? ("Denken Sie, dass die Themen "Klimaschutz", "Energiewende" und "Nachhaltigkeit" klinische Entscheidungen in Ihrer Abteilung in der Zukunft beeinflussen können?“)**


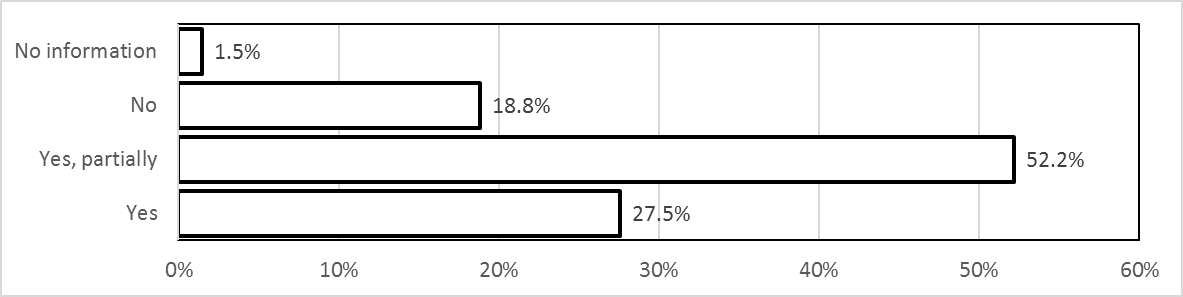


**Question 10: Would you welcome/promote the inclusion of such topics in everyday medical and radiotherapy practice? ("Würden Sie es begrüßen/fördern, wenn solche Themen mehr Einzug in den medizinischen und strahlentherapeutischen Alltag finden würden?")**


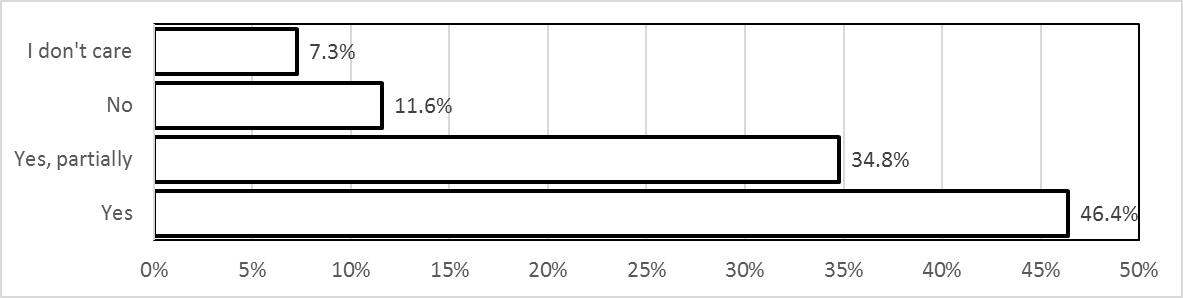


***Question 11: In which areas should it be used more? („In welchen Bereichen sollte es mehr Einzug finden (freie Nennung)?“)***

**Technical measures Buildings**

**(„Technische Maßnahmen Gebäude“)**

**-----------------**

**Generating electricity from sustainable sources**

**(„Stromgewinnung aus nachhaltigen Quellen“)**

**-----------------**

**Due to national and international obligations such as the Paris Climate Agreement, the Supply Chain Act and the national circular economy strategy, sustainability must be implemented in all areas. Otherwise it will become expensive at some point. If not for us, then for our descendants.**

**(„Wegen nationaler und internationaler Verpflichtungen wie z.B. dem Pariser Klima-Abkommen, dem Lieferkettengesetz und der nationalen Kreislaufwirtschaftsstrategie muss Nachhaltigkeit in allen Bereichen Einzug finden. Sonst wird es irgendwann teuer. Wenn nicht für uns, dann für unsere Nachkommen.“)**

**-----------------**

**Digitization**

**(„Digitalisierung“)**

**-----------------**

**Renewable energies, low power consumption in standby mode, energy-saving concepts**

**(„erneuerbare Energien, wenig Stromverbrauch im Standby, energieschonende Konzepte“)**

**-----------------**

**Commuting, switching off appliances and lights overnight.**

**(„Arbeitsweg, Ausschalten von Geräten und Licht über Nacht.“)**

**-----------------**

**Utilization of waste heat from large appliances, reduction of standby times, reduction of packaging and disposable items**

**(„Nutzung von Abwärme bei Großgeräten, Reduktion von standby-Zeiten, Reduktion von Verpackungen und Einwegartikeln“)**

**-----------------**

**Nowhere. “Man-made climate change” is above all a construct of global elites and has not been scientifically proven.**

**(„Nirgends. "Menschengemachter Klimawandel" ist vor allem ein Konstrukt globaler Eliten und wissenschaftlich nicht stichhaltig bewiesen.“)**

**-----------------**

**Handling disposable materials (inpatient and outpatient)**

**(„Umgang mit Einweg-Material (stationär und ambulant)“)**

**-----------------**

**Use waste heat from the linacs, reduce lighting if necessary, automatic PC shutdown at night**

**(„Abwärme der Linacs nutzen, Beleuchtung ggf reduzieren, automatische PC Abschaltung in den Nachtstunden“)**

**-----------------**

**Device Selection („Geräteauswahl“)**

**Treatment concepts („Behandlungskonzepte“)**

**-----------------**

**Saving energy is a must and will become an integral part of everyday clinical practice.**

**(“Energieeinsparung ist ein Muss und wird sich in den klinischen Alltag drängen.“)**

**-----------------**

**Recommendation of radiotherapeutic concepts (number of fractions, at least in the case of equieffectiveness, take the protocol that is less harmful to the climate, i.e. number of fractions in particular, keyword hypofractionation, particle therapy only if absolutely and clearly better/superior).**

**(„Empfehlung von strahlentherapeutischen Konzepten (Anzahl der Fraktionen, zumindest bei Äquieffektivität das Protokoll nehmen, das weniger klimaschädlich ist, also Anzahl Fraktionen insbesondere, Stichwort Hypofraktionierung, Partikeltherapie nur, wenn unbedingt und deutlich besser/überlegen).“)**

**-----------------**

**IT - Shutting down computers at the end of the working day („IT - Rechner herunterfahren bei Dienstschluss“)**

**evidence-based hypofractionation („evidenzbasierte Hypofraktionierung“)**

**General energy-saving measures in clinics („Generelle Energieeinsparmaßnahmen in Kliniken“)**

**Check consumables („Verbrauchsmaterialien prüfen“)**

**Use of medication („Medikamenteneinsatz“)**

**-----------------**

**Operation of large medical equipment, outpatient radiotherapy care (avoidance of individual trips to therapy)**

**(„Betrieb von medizinischen Großgeräten, ambulante strahlentherapeut. Versorgung (Vermeidung individueller Einzelfahrten zur Therapie)“)**

**-----------------**

**Digitization instead of paper: Around 2600 kWh are needed to produce one tonne of paper. In addition, CO2 is produced during transportation, delivery and waste disposal. And the paper industry destroys tree populations that absorb CO2. As more trees are cut down worldwide than are reforested, this also contributes to the deterioration of the CO2 balance.**

**If you want to check how good/bad your clinic is in this respect:**

**https://www.papiernetz.de/informationen/nachhaltigkeitsrechner/**

**Incidentally, digitalization is also worthwhile for optimizing work processes in terms of time.**

**(„Digitalisierung statt Papier: Für die Produktion von einer Tonne Papier werden ca. 2600 kWh benötigt. Dazu entsteht noch CO2 beim Transport während der Anlieferung und Abfallentsorgung. Und die Papierindustrie vernichtet Baumbestände, die CO2 aufnehmen. Da weltweit mehr abgeholzt als aufgeforstet wird, trägt auch das zur Verschlechterung der CO2-Bilanz bei.**

**Wenn Sie prüfen wollen, wie gut/schlecht Ihre Klinik diesbezüglich ist:**

**https://www.papiernetz.de/informationen/nachhaltigkeitsrechner/**

**Übrigens lohnt sich Digitalisierung auch zur zeitlichen Optimierung der Arbeitsabläufe.“)**

**-----------------**

**Heating management, lighting management, new building management, employee training, employee awareness.**

**(„Heizmanagement, Lichtmanagement, Neubaumanagement, Mitarbeiterschulungen, Bewusstsein der Mitarbeiter.“)**

**-----------------**

**Digitalization, use of renewable energies in-house, shorter therapy times**

**(„Digitalisierung, erneuerbare Energien nutzen im Hause, kürzere Therapiezeiten“)**

**-----------------**

**Digitization? („Digitalisierung?“)**

**Patient transportation („Patiententransporte“)**

**-----------------**

**Savings on heating („Einsparung Heizung“)**

**-----------------**

**raise awareness in the first place or seriously address identified issues. My answer to question 8 refers to a “flash in the pan” at the time of the gas crisis at the beginning of the Ukraine conflict. When the immediate danger was over, little or nothing happened again and my impression is that there is no personnel and possibly also no capital for the high initial investments (e.g. photovoltaics,...).**

**Something also needs to happen with the manufacturers / in the procurement process. The topic is virtually not being addressed and the critical trade-off between “uptime/availability/safe operation” vs. energy saving (why do devices have to stay switched on 24/7 if you only work with them for 10 hours) needs to be discussed.**

**(„überhaupt eine Sensibilisierung schaffen bzw. identifizierte Themen ernsthaft angehen. Meine Antwort zu Frage 8 bezieht sich auf ein "Strohfeuer" zu Zeiten der Gaskriesen zu Beginn des Ukraine Konflikts. Als die unmittelbare Gefahr vorbei war, ist wieder wenig bis nichts passiert und mein Eindruck ist, dass es kein Personal und ggf. auch kein Kapital gibt für die hohen Anfangsinvestitionen (zB Photovoltaik,...).**

**Auch bei den Herstellern / im Vergabeverfahren muss etwas passieren. Das Thema wird quasi nicht thematisiert und die kritische Abwägung "Uptime/Verfügbarkeit/sicheres Betreiben" vs. Energiesparen (warum müssen Geräte 24/7 angeschaltet bleiben, wenn man nur 10h damit arbeitet) muss besprochen werden.“)**

**-----------------**

**Energy recovery from cooling and air conditioning technology**

**(„Energierückgewinnung aus Kühlungs- und Klimatechnik“)**

**-----------------**

**- Number of fractions („Fraktionsanzahl“)**

**- Remuneration, as you are not rewarded for more fractions (if not necessary)**

**(„Vergütung, da einen nicht für mehr Fraktionen (wenn nicht nötig) belohnt“)**

**- Purchase of devices („Anschaffung von Geräten“)**

**- Erneuerungszyklen von Stromverbrauchern (dazu zählen auch PCs)**

**(„Renewal cycles of power consumers (including PCs)“)**

**- Manufacture of the medical devices used**

**(„Herstellung der verwendeten Medizinprodukte“)**

**-----------------**

**Station/Consumables („Station/Verbrauchsmaterialien“)**

**-----------------**

**The second question is absolute nonsense and has zero relevance!!!!**

**It should be used sensibly: Urban lighting or promoting public transportation.**

**However, this topic has no place in radiotherapy. („Die zweite Frage ist absoluter Quatsch und hat null Relevanz!!!!**

**Es sollte sinnvoll eingesetzt werden: Städtische Beleuchtung oder Förderung von Öffentlichen Verkehrsmitteln.**

**Dieses Thema hat jedoch nichts in der Strahlentherapie verloren.“)**

**-----------------**

**Resources, consumption, saving electricity and energy, more use in energy production**

**(„Ressourcen , Verbrauch, sparen Strom und Energien, mehr Einsatz in Gewinnung von Energien“)**

**-----------------**

**Patient logistics, therapy route optimization**

**(„Patientenlogistik, Therapiewegeoptimierung“)**

***Question 12: How much energy do you estimate your linear accelerator requires per day? For comparison: A refrigerator requires around 0.2 kWh per day [6]. (“Was schätzen Sie, wie viel Energie Ihr Linearbeschleuniger pro Tag an Energie benötigt? Zum Vergleich: Ein Kühlschrank benötigt pro Tag circa 0,2 kWh [6].“)***

***
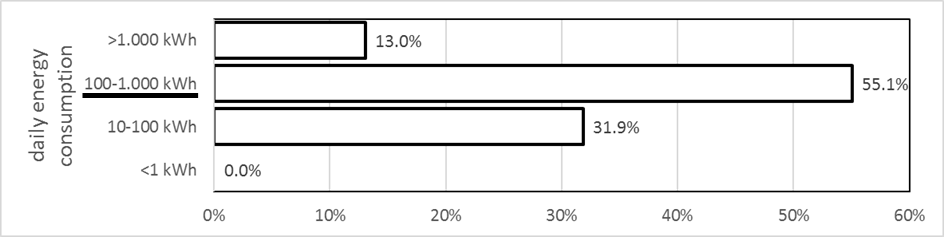
***

***Question 13: Does your linear accelerator have an energy-saving “sleep” mode? (“Verfügt Ihr Linearbeschleuniger über einen energiesparenden „Sleep“-Modus?“)
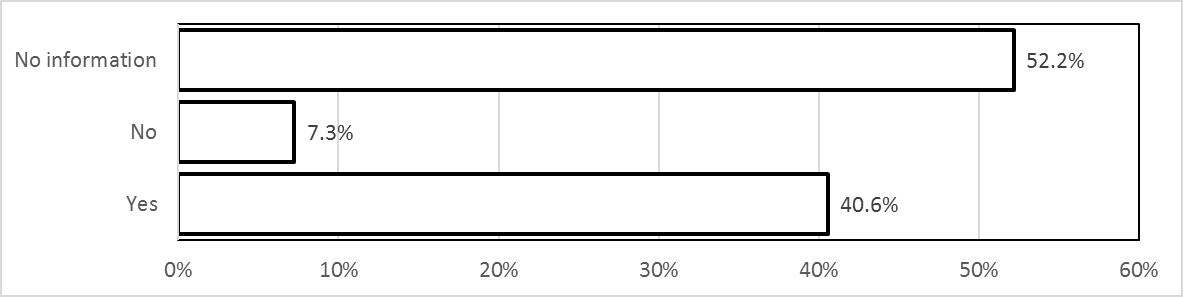
***

***Question 14: Is it important to you that linear accelerator manufacturers produce sustainably or that the service/maintenance work demonstrates sustainable aspects? (“Ist es Ihnen wichtig, dass Linearbeschleunigerhersteller nachhaltig produzieren respektive die Service- und Wartungsarbeiten nachhaltige Aspekte aufweisen?“)***

***
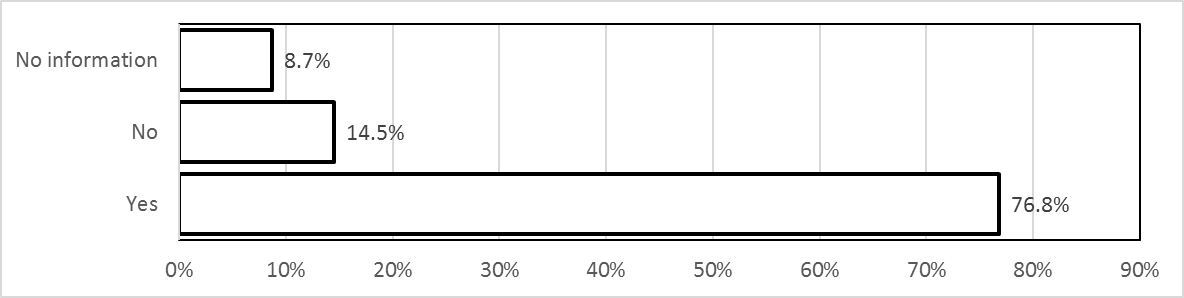
***

***Question 15: Would that be a decision criterion for a new purchase? (“Wäre das ein Entscheidungskriterium bei einer Neuanschaffung?“)***

***
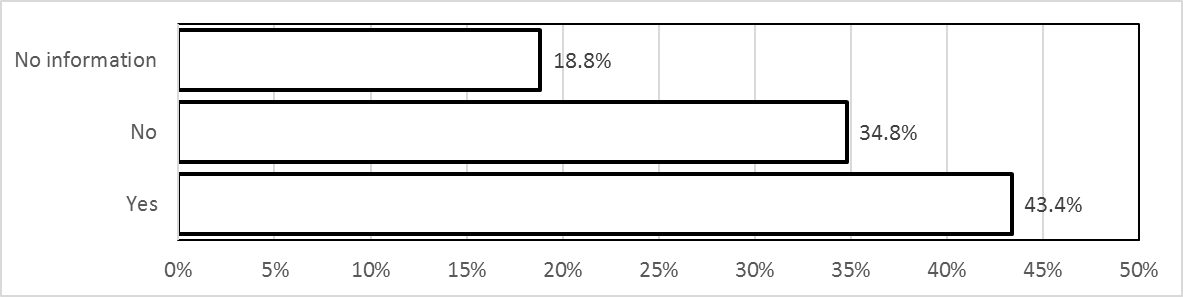
***

***Question 16:*** ***Are you thinking about investing in an energy meter to quantify/optimize your energy consumption at the linear accelerator? (“Denken Sie darüber nach, in ein Energiemessgerät zu investieren, um Ihren Energieverbrauch am Linearbeschleuniger zu quantifizieren/optimieren?“)***

***
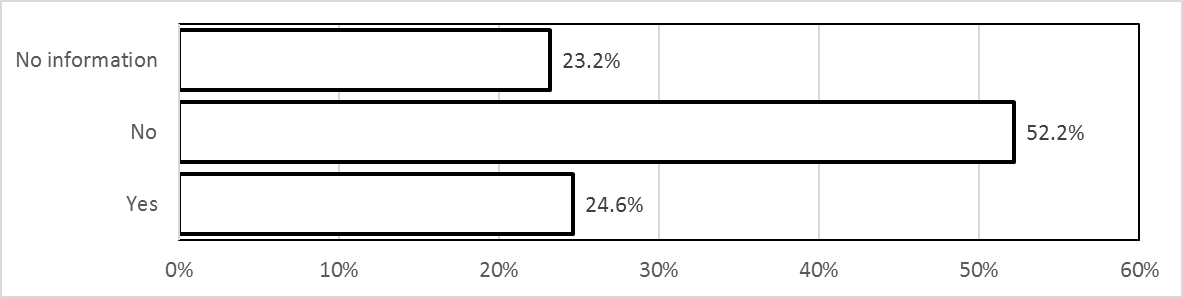
***

***Question 17: When creating/optimizing plans, would you pay attention to the generation of power/time-saving variants (e.g. fewer rotation fields in VMAT plans)? (“Würden Sie bei der Planerstellung und –optimierung auf die Generierung von strom-/zeitsparenden Varianten achten (z.B. weniger Rotationsfelder bei VMAT-Plänen)?“)***

***
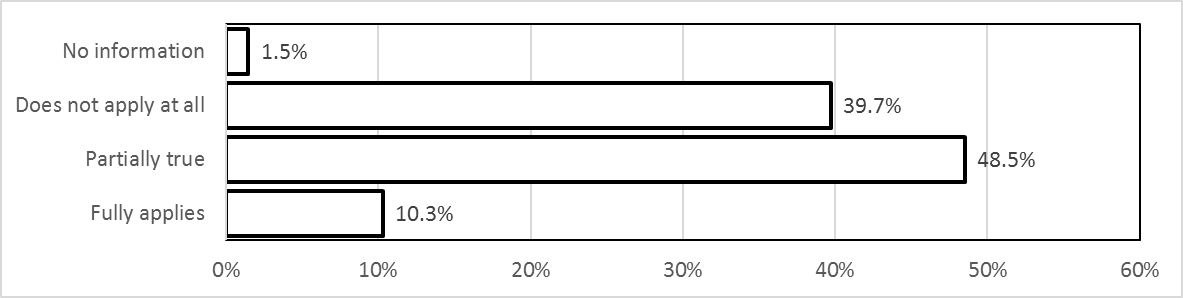
***

***Question 18: How do you assess the current development towards more climate-neutral processes in the healthcare sector (“DGMP goes green”, “ESTRO Climate Change Awareness and Action”, “KITTEN, ‘KlinKe’)? (“Wie beurteilen Sie die aktuelle Entwicklung zu klimaneutraleren Prozessen im Gesundheitswesen ("DGMP goes green", "ESTRO Climate Change Awareness and Action", "KITTEN", "KlinKe")?“)***

***
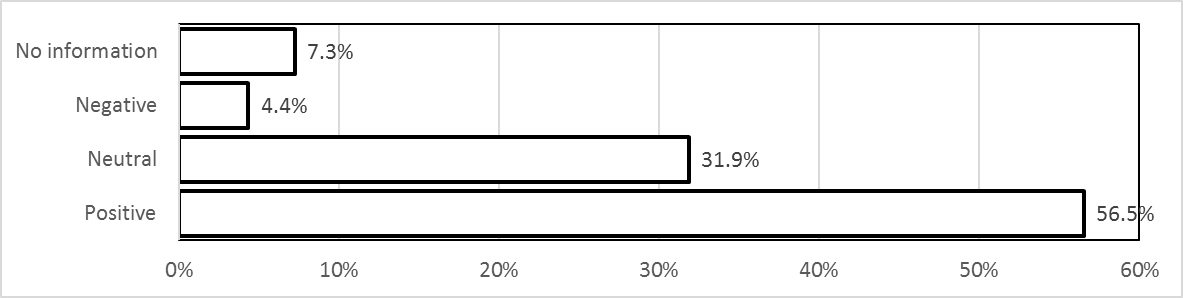
***

***Question 19: Do you use ultrahypofractionated radiation concepts (single dose 5 Gy or more) in your department? (“Verwenden Sie in Ihrer Abteilung ultrahypofraktionierte Bestrahlungskonzepte (Einzeldosis 5 Gy oder mehr)?“)***

***
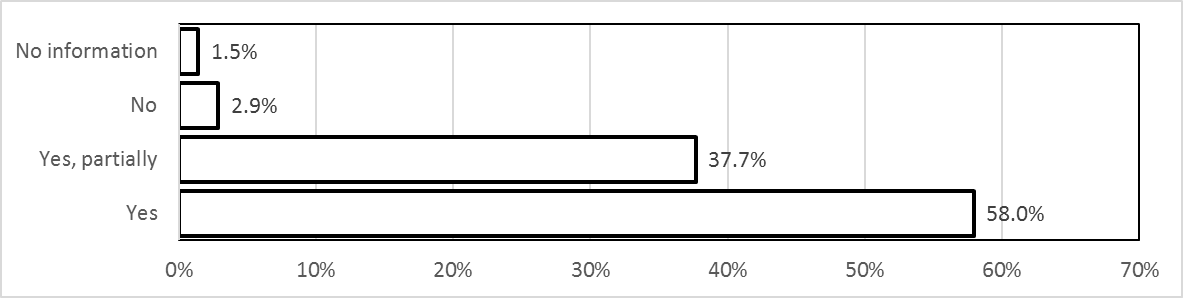
***

***To question 19: If yes, for what reasons? (multiple answers possible) („Falls ja, aus welchen Gründen? (Mehrfachnennungen möglich)“)***

***
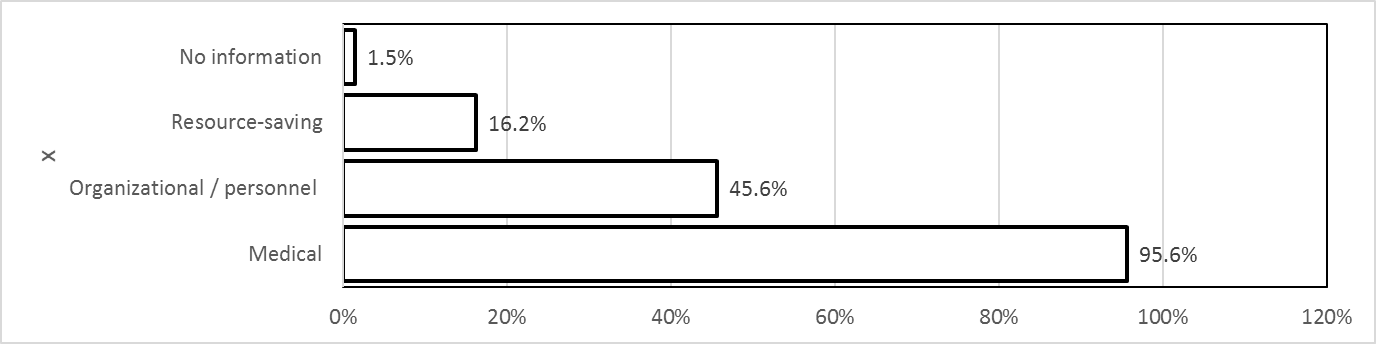
***

***Question 20:*** ***Which fractionation scheme is the current standard in your radiotherapy facility for breast cancer (without sequential boost, without irradiation of the lymphatic drainage pathways)? (“Welches Fraktionierungsschema stellt den gegenwärtigen Standard in Ihrer strahlentherapeutischen Einrichtung beim Mammakarzinom dar (ohne sequentiellen Boost, ohne Bestrahlung der Lymphabflußwege)?”)***

***
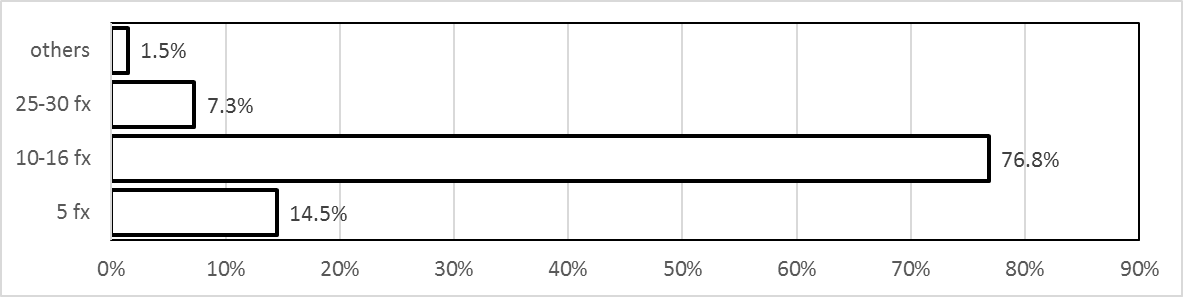
***

***Question 21: Which fractionation scheme is the current standard in your radiotherapy facility for prostate cancer (without irradiation of the pelvic lymphatic drainage pathways)? (“Welches Fraktionierungsschema stellt den gegenwärtigen Standard in Ihrer strahlentherapeutischen Einrichtung beim Prostatakarzinom (ohne Bestrahlung der pelvinen Lymphabflußwege) dar?“)***

***
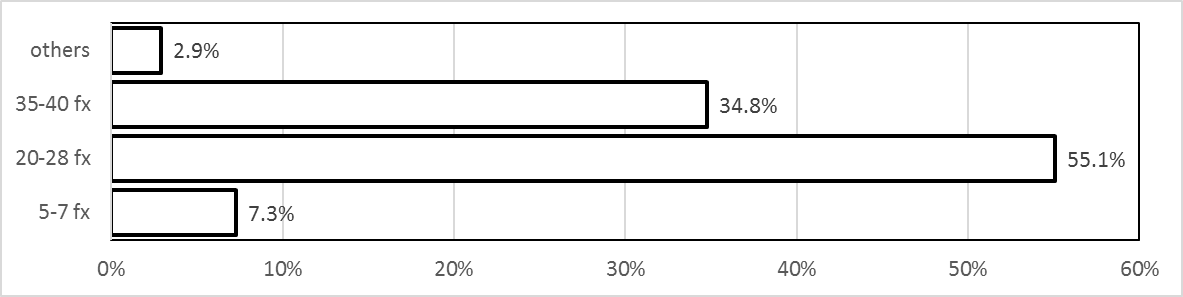
***

***Question 22: Which fractionation scheme is most frequently used in your radiotherapy facility for bone metastases? (“Welches Fraktionierungsschema wird am häufigsten in Ihrer strahlentherapeutischen Einrichtung bei Knochenmetastasen eingesetzt?“)***

***
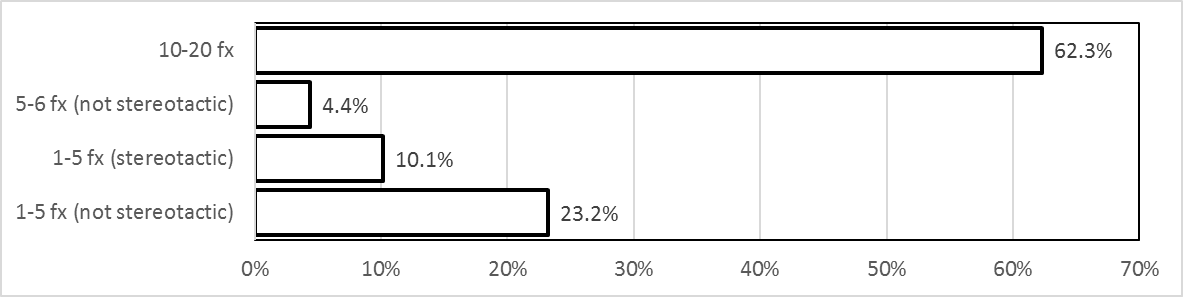
***

***Question 23: To what extent do you think normofractionation increases the CO_2_ footprint compared to ultra-hypofractionation (e.g. 28 fractions versus 5 fractions)? (“Was denken Sie, inwieweit Normofraktionierung im Vergleich zu Ultra-Hypofraktionierung den CO_2_-Fußabdruck erhöht (z.B. 28 Fraktionen versus 5 Fraktionen)?“)***

***
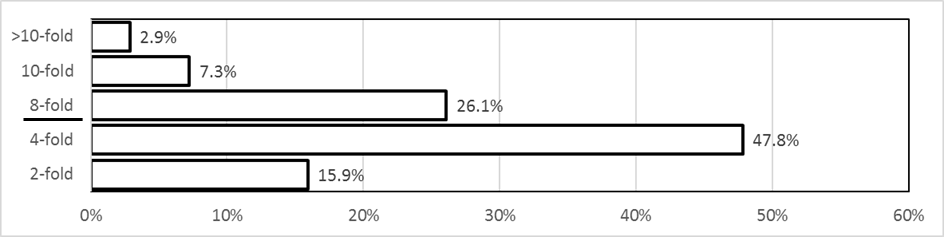
***

***Question 24: Would you increasingly switch to ultrahypofractionated concepts with fewer fractions if there were no medical disadvantages for patients (no poorer tumor control, no more significant side effects)? (“Würden Sie verstärkt auf ultrahypofraktionierte Konzepte mit weniger Fraktionen umstellen, wenn dadurch keine medizinischen Nachteile (keine schlechtere Tumorkontrolle, nicht mehr signifikante Nebenwirkungen) für die Patienten entstehen?“)***

***
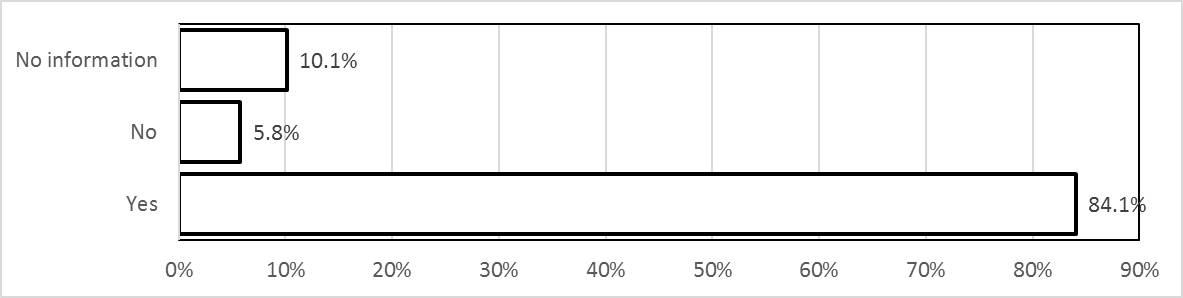
***

***Question 25: A patient with prostate cancer drives 20 km by car from his home to the radiotherapy center. In your opinion, how much CO_2_ can be saved by a hypofratcionized concept (7 trips instead of 39)? (“Ein Patient mit Prostatakarzinom fährt mit seinem PKW von seinem Wohnort bis zur Strahlentherapie 20 km. Wie viel CO_2_ kann man Ihrer Einschätzung nach durch eine hypofraktioniertes Konzept einsparen (7 Fahrten anstatt 39 Fahrten)?“)***

*
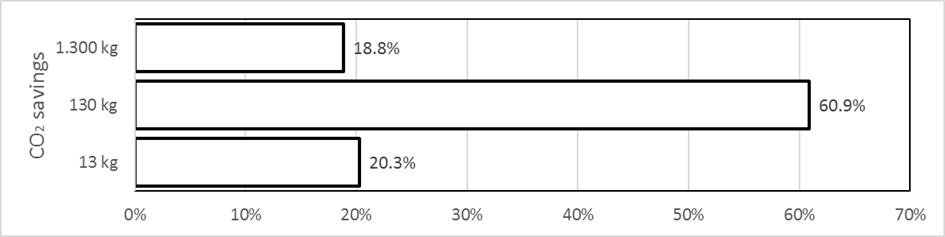
*

In the text field below you have the opportunity to give us free comments and advice on the topic of this survey. Thank you very much for your participation! („Im nachfolgenden Textfeld haben Sie die Möglichkeit uns freie Anmerkungen und Hinweise zum Thema dieser Umfrage zu geben. Herzlichen Dank für Ihre Teilnahme!“)

-----------------

In my opinion, the medical aspects will/should always come first in radiotherapy! („In der Strahlentherapie werden/sollten nach meiner Einschätzung immer die medizinischen Aspekte unangefochten an erster Stelle stehen!“)

-----------------

It's great that there are people who take responsibility and think outside the box. („Schön, dass es Leute gibt, die Verantwortung übernehmen und über ihren Tellerrand hinaus schauen.“)

-----------------

Exciting survey that sheds light on current topics. („Spannende Umfrage, die aktuelle Themen beleuchtet.“)

-----------------

This survey is very useful for gaining an overview of how high the burden of operating linear accelerators actually is, especially in comparison to other radiotherapy departments in Germany. The exchange of such data can provide valuable insights into how efficiently different facilities work and where there is potential for optimization. („Diese Umfrage ist durchaus sinnvoll, um sich einen Überblick darüber zu verschaffen, wie hoch die Belastung durch den Betrieb von Linearbeschleunigern tatsächlich ist, insbesondere im Vergleich zu anderen Strahlentherapieabteilungen in Deutschland. Der Austausch solcher Daten kann wertvolle Erkenntnisse darüber liefern, wie effizient verschiedene Einrichtungen arbeiten und wo Optimierungspotenzial besteht.“)

-----------------

Why are you asking for so many personal details? Then asking for the federal state unfortunately allows conclusions to be drawn about the person and removes the anonymization. Hence the federal state “other”. („Warum erfragen Sie so viele Details zur Person? Dann noch nach dem Bundesland fragen lässt leider auf die Person schlussfolgern und hebt die Anonymisierung auf. Daher Bundesland "sonstige".“)

-----------------

Green ideology has no place in our field. However, you could set a good example and suggest to the Rhön-Klinikum that the proton center in Marburg be shut down - you would immediately have massive energy-saving potential. At least be consistent in your efforts! („Grüne Ideologie hat in unserem Fach nichts zu suchen. Sie könnten allerdings mit gutem Beispiel vorangehen und dem Rhön-Klinikum vorschlagen, das Protonenzentrum in Marburg stillzulegen - da hätten Sie sofort ein massives Energie-Einsparpotenzial. Seien Sie dann wenigstens konsequent in ihren Bemühungen!“)

-----------------

At no point in your survey is the necessary daily balancing act between billing, guidelines and now also energy saving addressed, I am glad that these topics are discussed, but it is a multifactorial process („in Ihrer Befragung wird an keiner Stelle auf den täglichen notwendigen Spagat zwischen Abrechung, Leitlinie und nun auch noch zusätzlich Energieeinsparung eingegangen, ich bin froh, dass diese Themen besprochen werden, aber es ist halt ein multifaktorielles Geschehen“)

-----------------

food for thought

-----------------

Work in Upper Austria, but is not listed, therefore 'Bavaria' selected („Arbeite in Oberösterreich, ist aber nicht gelistet, daher ‚Bayern‘ ausgewählt“)

-----------------

Keep up the good work! („Weiter so!“)

-----------------

DEGRO still refuses to deliver the journal Strahlentherapie und Onkologie only digitally and no longer in print. Print cannot be discontinued. The reason is advertising contracts. This is completely out of date. Before such surveys are conducted, simple steps should be taken to conserve resources. („Die DEGRO weigert sich nach wie vor, die Zeitschrift Strahlentherapie und Onkologie nur noch digital und nicht mehr als Print zuzustellen. Man kann den Print nicht abbstellen. Grund sind Werbeverträge. Das ist absolut aus der Zeit gefallen. Bevor man solche Umfragen schaltet, sollte man erstmal die ganz einfachen Schritte zur Ressourcenschonung umsetzen.“)

-----------------

Interesting survey, I'd like to see more. („Interessante Umfrage, gerne mehr davon.“)

-----------------

Sustainability is important. Nevertheless, it should never take precedence over medically necessary therapies (a 4-field box may be more energy-efficient than 2 full rotations). In general, more attention should be paid to this in the medical field, but rather in the environment. I don't think it's right to accept poorer treatments (possibly with hypofractionation) in order to save energy, and this may not be accepted by many. Furthermore, it will not help to set billing options against potential energy savings. This would require a fundamental reform of billing. Otherwise, it will unfortunately also meet with little acceptance. A good approach would also be for centers to produce more of their own electricity and for employees to pay more attention to potential savings. („Nachhaltigkeit ist wichtig. Dennoch sollte es nie Vorrang gegenüber medizinisch notwendigen Therapien haben (ggf. ist eine 4-Felder Box energiesparender als 2 Vollrotationen). Generell im Medizinbereich sollte darauf stärker geachtet werden, aber eher im Umfeld. Schlechtere Behandlungen zu akzeptieren (ggf. auch mit Hypofraktionierung), um dadurch Energie zu sparen halte ich nicht für richtig und wird ggf. auch an der Akzeptanz vieler scheitern. Des Weiteren wird es auch nichts bringen, Abrechnungsmöglichkeiten gegen Energieeinsparpotenziale zu setzen. Hierzu müsste eine grundlegende Reform der Abrechnung stattfinden. Ansonsten wird es leider auch auf wenig Akzeptanz stoßen. Ein guter Ansatz wäre es auch, wenn Zentren vermehrt ihren Strom selbst produzieren und Mitarbeiter selbst eher auch auf Einsparungsmöglichkeiten achten.“)

-----------------

It would be nice to have a solution to the “quiz questions”. How big is the savings potential? Have I estimated correctly or am I completely wrong? („Schön wäre eine Auflösung der "Quizfragen". Wie groß ist den das Einsparpotential? Habe ich richtig eingeschätzt oder liege ich völlig daneben?“)

-----------------

frightening how little the individual is still worth to us...(„erschreckend wie wenig uns das Individuum noch wert ist...“)

-----------------

Medical issues should have priority. („medizinische Fragestellungen sollten Vorrang haben.“)

-----------------

Very nice survey, Daniel! The average number of fractions - averaged across all indications - is a nice benchmark for the degree of hypofractionation. We are currently at 14.5... incl. the few benign ones then at 12.5... („Sehr schöne Umfrage, Daniel! Die durchschnittliche Anzahl der Fraktionen - auf alle Indikationen gemittelt - ist ein schöner Benchmark für den Grad an Hypofraktionierung. Wir liegen aktuell bei 14,5... incl. den paar benignen dann bei 12,5... „)

-----------------

F23 and 25, among others, are imprecise. Does F23 include everything (incl. travel, preliminary examination, planning, ...) or only the individual radiation treatments? F24 includes CO2 impression as well as billing, ... so that more detailed information would be necessary to answer them. („u.a. F23 und 25 sind ungenau gestellt. Beinhaltet F23 alles (inkl Anreise, Voruntersuchung, Planung, ...) oder nur die einzelnen Bestrahlungen. F24 beinhaltet neben CO2 Abdruck auch Abrechnung, ... so dass nähere Angaben notwendig wären, um sie zu beantworten.“)

-----------------

Against the background of therapy costs in the high four-digit range per month for immunotherapy or targeted therapy, I consider the focus on saving a few kWh per patient to be an absurd consideration from a macroeconomic point of view. One pack of osimertinib corresponds to the cost of more than 20000 kWh. („Vor dem Hintergrund von Therapiekosten im hohen vierstelligen Bereich pro Monat für Immun- oder targeted Therapie halte ich den Fokus auf Einsparung von einigen kWh pro Patient für eine gesamtwirtschaftlich absurde Überlegung. Eine Packung Osimertinib entspricht den Kosten von mehr als 20000 kWh.“)

-----------------

I don't really care about co2. („Eigentlich ist mir co2 egal“)

-----------------

The current attitude to the subject is suspect simply because of the way your questions are formulated. This is not about feeling good about a low-energy VMAT or the journey to the next treatment option, but solely about the medical quality of the radiation. („Die aktuelle Einstellung zu dem Thema ist allein durch die Formulierung Ihrer Fragen suspekt. Hier geht es nicht um ein gutes Gefühl bei einer energiearmen VMAT oder den Fahrtweg zur nächsten Therapiemöglichkeit, sondern einzig und allein um die medizinische Qualität der Bestrahlung.“)

-----------------

Exciting and hopefully something will change, good suggestions coming together („Spannend und hoffentlich etwas verändern, gute Vorschläge dazu zsusammen kommend“)
